# Methylene Blue Alleviates Inflammatory and Oxidative Lung Injury in a Rat Model of Feces-Induced Peritonitis

**DOI:** 10.3390/medicina61081456

**Published:** 2025-08-13

**Authors:** Cengiz Dibekoğlu, Kubilay Kemertaş, Hatice Aygun, Oytun Erbas

**Affiliations:** 1 Department of General Surgery, Demiroğlu Bilim University, Istanbul 34000, Turkey; cdibekoglu@gmail.com; 2 Department of General Surgery, Florence Nightingale Hospital, Istanbul 34000, Turkey; drkemertas@gmail.com; 3 Neuroscience Laboratory, BAMER, Biruni University, Istanbul 34000, Turkey; 4 Faculty of Medicine, BAMER, Biruni University, Istanbul 34000, Turkey; oytunerbas2012@gmail.com

**Keywords:** sepsis, feces-induced peritonitis, methylene blue, acute lung injury, inflammation, oxidative stress, cyclic guanosine monophosphate

## Abstract

*Background and Objectives:* Feces-induced peritonitis (FIP), a clinically relevant model of polymicrobial sepsis, induces systemic inflammation and acute lung injury (ALI). Methylene blue (MB), a phenothiazine-based compound, exhibits vasoregulatory, antioxidant, and anti-inflammatory properties in the context of sepsis. This study aimed to evaluate the protective effects of MB on pulmonary injury in a rat model of FIP-induced sepsis. *Materials and Methods:* Forty male Wistar rats were randomly assigned to four groups: control, FIP, FIP + Saline, and FIP + MB. MB was administered intraperitoneally at a dose of 20 mg/kg, 1 h after FIP induction. At 24 h post-induction, plasma levels of inflammatory markers [interleukin-6 (IL-6), interleukin-1 beta (IL-1β), tumor necrosis factor-alpha (TNF-α), C-reactive protein (CRP)], oxidative stress marker [malondialdehyde (MDA)], metabolic indicator [lactic acid], and vascular signaling marker [cyclic guanosine monophosphate (cGMP)] were measured. Lung injury was evaluated through histopathological analysis and thoracic computed tomography (CT)-based Hounsfield unit (HU) quantification, while pulmonary function was assessed via arterial blood gas analysis, including arterial oxygen pressure (PaO_2_) and carbon dioxide pressure (PaCO_2_). *Results:* FIP induction led to significant increases in plasma levels of IL-6, IL-1β, TNF-α, CRP, MDA, cGMP, and lactic acid, accompanied by elevated CT attenuation (HU) values and a marked reduction in arterial PaO_2_ and PaCO_2_. MB treatment significantly decreased the levels of IL-6, IL-1β, TNF-α, CRP, MDA, lactic acid, and cGMP, improved PaO_2_, and attenuated both histopathological lung injury and CT-assessed parenchymal density. No significant differences were observed in PaCO_2_ among the groups. *Conclusions:* MB mitigates inflammation, oxidative damage, and pulmonary dysfunction in FIP-induced sepsis. Further studies are warranted to optimize dosing and timing and to evaluate long-term outcomes.

## 1. Introduction

Sepsis remains a major global health burden, affecting approximately 240–300 individuals per 100,000 annually in the United States alone [1,2]. One of its most serious complications, sepsis-associated acute lung injury (ALI), and its advanced form, acute respiratory distress syndrome (ARDS), markedly worsen clinical outcomes [3]. Despite advances in supportive care and antimicrobial therapy, effective pharmacological interventions are still limited due to the complex pathophysiology of sepsis-induced ALI. Key mechanisms include endothelial damage, cytokine-driven inflammation, oxidative stress, mitochondrial dysfunction, dysregulated nitric oxide (NO) signaling, and pulmonary microvascular barrier disruption [4]. Among adjunctive therapies, methylene blue (MB)—a phenothiazine dye with established clinical use—has gained attention for its favorable safety profile and multi-targeted mechanisms [5].

MB’s therapeutic potential in sepsis is largely attributed to its inhibition of the NO–soluble guanylate cyclase (sGC)–cyclic guanosine monophosphate (cGMP) pathway. Excessive NO production via inducible NO synthase (iNOS) leads to vasoplegia and tissue hypoperfusion due to elevated cGMP levels [6,7,8]. MB inhibits both iNOS and sGC, reducing cGMP levels, restoring vascular tone, and improving vasopressor responsiveness [5,9,10]. As direct NO measurement in vivo is technically limited due to its short half-life [11,12], cGMP serves as a reliable surrogate marker of NO–sGC activation [13,14,15]. Accordingly, plasma cGMP was selected in this study to assess the pharmacodynamic effects of MB.

Beyond its vasoregulatory role, MB exhibits significant antioxidant and anti-inflammatory effects in diverse preclinical models of acute lung injury (ALI). Experimental studies in contexts such as sepsis, trauma, ischemia–reperfusion, and toxin-induced injury consistently show that MB mitigates oxidative stress and inflammation while preserving pulmonary architecture [10,16,17,18,19,20]. However, its pulmonary effects in polymicrobial sepsis models remain insufficiently characterized. In cecal ligation and puncture (CLP) models, Pan et al. [9] reported improved hemodynamic parameters through NO–cGMP inhibition, yet cytokine suppression was minimal. Likewise, Mestriner et al. [21] observed enhanced survival and reduced leukocyte adhesion without evaluating lung-specific outcomes. Among limited studies, only Demirbilek et al. [16] clearly demonstrated MB-mediated improvements in lung histopathology and oxidative markers in sepsis. Despite these findings, the effects of methylene blue (MB) in the fecal intraperitoneal injection (FIP) model, a more severe and standardized model of polymicrobial sepsis, have not been adequately investigated, highlighting the need for further focused research.

The FIP model induces a more severe and consistent septic insult compared to CLP, another widely used polymicrobial sepsis model, with stronger cytokine responses and prominent lung injury, including alveolar destruction and hemorrhage within 24 h [22]. Its use of standardized fecal slurry enhances reproducibility and reduces variability in disease severity [22,23]. Unlike CLP, where results vary by technique and microbiota, FIP yields more uniform inflammation and organ dysfunction [23,24]. In this study, the FIP model offered a relatively consistent septic challenge across animals, supporting a more standardized evaluation of MB’s pulmonary effects. Given its capacity to induce robust septic ALI, FIP may serve as a suitable platform for investigating lung-targeted therapies.

In the present study, we aimed to elucidate the potential protective effects of MB against sepsis-induced acute lung injury using the feces intraperitoneal injection (FIP) model. To achieve this, we integrated biochemical, histopathological, and radiological assessments under conditions of severe polymicrobial insult.

## 2. Materials and Methods

### 2.1. Animals

A total of 40 healthy adult male Wistar albino rats (250–300 g) were used, obtained from the Experimental Animal Research Facility of Science University. Animals were maintained under a 12 h light/dark cycle at a constant temperature of 23–25 °C, with ad libitum access to standard chow and tap water. All procedures complied with the NIH Guide for the Care and Use of Laboratory Animals and were approved by the Science University Animal Research Ethics Committee (approval No.: 02210623).

Sample sizes for each group (*n* = 9–10) were initially determined based on previously published feces-induced peritonitis (FIP) models in rodents that reported large treatment effects across inflammatory, oxidative, and histopathological outcomes [25,26,27]. This study design adheres to the 3Rs ethical principle (Replacement, Reduction, and Refinement), aiming to minimize animal use while ensuring scientific validity.

### 2.2. Experimental Protocol

#### 2.2.1. Sepsis Induction and Experimental Design

To establish an experimental sepsis model, adult male Wistar albino rats were randomly assigned to four experimental groups. A total of 40 rats were included in the study, of which 30 underwent sepsis induction via the FIP method, while 10 rats were assigned to the normal control group without any invasive intervention. The FIP-induced sepsis model was performed in accordance with the protocol described by Sever et al. [25], with minor modifications.

A single intraperitoneal (i.p.) dose of MB (20 mg/kg) was selected based on prior studies demonstrating its antioxidant and hemodynamic efficacy without toxicity in rodents as well as its effectiveness in reducing sepsis-induced lung injury [16,28,29]. MB was administered immediately after FIP induction to target the hyperinflammatory phase, consistent with established protocols favoring early intervention for optimal therapeutic benefit [16,25].

Isotonic saline (0.9% NaCl) was selected as the placebo because it is physiologically inert, commonly used for intraperitoneal administration, and was also the solvent in which MB was dissolved, providing an appropriate control for injection volume and vehicle effects [16,25].

#### 2.2.2. Fecal Slurry Preparation and Sepsis Induction

Fresh feces samples were collected from donor rats and homogenized in sterile 0.9% saline to prepare a fecal suspension. The final concentration was adjusted to 1 g of feces per 1 mL of saline. This fecal slurry was administered intraperitoneally at a dose of 1 g/kg body weight using a sterile syringe under mild anesthesia. This procedure was applied to induce polymicrobial sepsis in the experimental rats. Two rats died within the first 24 h post-injection and were excluded from the final analysis. All treatments were initiated 1 h after the FIP procedure, and the experimental duration was set at 24 h [22,25].

#### 2.2.3. Experimental Groups

The animals were divided into the following four groups (*n* = 8 per group, unless otherwise noted):

Group 1—Normal Control:

No surgical procedure or fecal slurry injection was performed.

Group 2—FIP Group (sepsis model):

Rats received an intraperitoneal injection of fecal slurry (1 g/kg) to induce sepsis. No further treatment was administered.

Group 3—FIP + Saline (placebo group):

Rats were subjected to the same FIP procedure as Group 2, followed by an intraperitoneal (i.p.) injection of 10 mL/kg/day isotonic 0.9% NaCl solution as a placebo treatment.

Group 4—FIP + Methylene Blue:

Following FIP induction, rats received 20 mg/kg/i.p. day of MB, administered once after 1 h.

To avoid confounding stress effects associated with injection procedures, no manipulation was applied to the normal control group. A separate FIP + Saline group was included to isolate the potential effects of vehicle injection from sepsis-induced alterations, thereby allowing a more accurate interpretation of treatment efficacy.

#### 2.2.4. Euthanasia and Sample Collection

**Figure 1:**
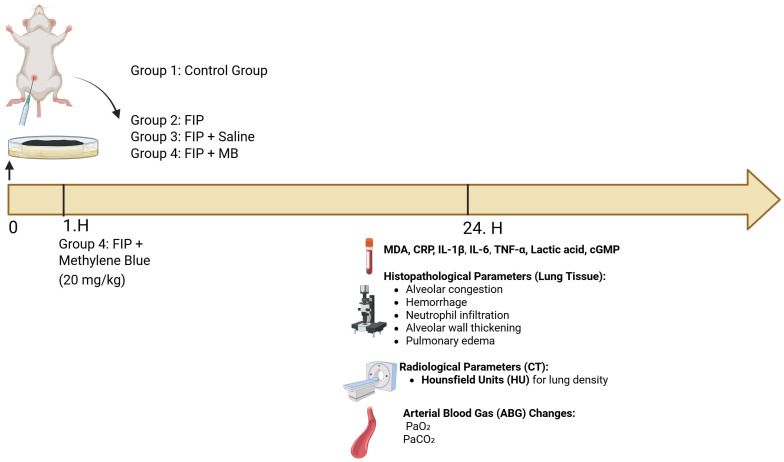
Experimental design and evaluated parameters in the FIP-induced sepsis model. A total of four groups were established: control (Group 1), feces-induced peritonitis (FIP, Group 2), FIP + Saline (Group 3), and FIP + MB (20 mg/kg, Group 4). FIP was induced via intraperitoneal fecal slurry administration. One hour after induction, MB was administered intraperitoneally to Group 4. All evaluations were conducted at the 24th hour post-induction. Biochemical markers (MDA, CRP, IL-1β, IL-6, TNF-α, lactic acid, cGMP), histopathological parameters (alveolar congestion, hemorrhage, neutrophil infiltration, alveolar wall thickening, pulmonary edema), radiological lung density via Hounsfield unit (HU) on CT, and arterial blood gas (PaO_2_ and PaCO_2_) measurements were performed.

At 24 h after FIP induction, all animals were anesthetized with an intraperitoneal injection of ketamine (40 mg/kg; Alfamine^®^, Alfasan International B.V., Woerden, The Netherlands) and xylazine (4 mg/kg; Alfazyne^®^, Alfasan International B.V., Woerden, The Netherlands). First, thoracic CT imaging was performed while the animals remained under deep anesthesia. Immediately afterward, 0.2 mL of arterial blood was collected from the right carotid artery using a heparinized syringe for arterial blood gas analysis (PaO_2_ and PaCO_2_). Subsequently, cardiac blood was obtained via direct puncture of the left ventricle with a 5 mL syringe for plasma biochemical assays. To ensure complete euthanasia in accordance with ethical standards, cervical dislocation was then performed as a secondary physical method. Following confirmation of death, the thoracic cavity was opened, and whole lung tissues were carefully excised for histopathological evaluations (Figure 1).

Cervical dislocation was performed as a secondary physical method following transcardial perfusion to ensure complete euthanasia in accordance with institutional and international ethical guidelines [30].

### 2.3. Histopathological Examination of Lung Tissue

#### 2.3.1. Anesthesia and Tissue Collection

Under deep anesthesia, thoracotomy was performed, and the animals were transcardially perfused with 200 mL of freshly prepared 4% formaldehyde solution in 0.1 M phosphate-buffered saline (PBS) to ensure optimal tissue fixation and vascular clearance.

#### 2.3.2. Fixation and Staining

The lungs were carefully excised and immersed in 10% neutral buffered formalin (NBF; 4% formaldehyde in phosphate buffer, pH ~7.0) for at least 48 h to preserve tissue structure and prevent acid-mediated autolysis. Following fixation, tissue samples were dehydrated, embedded in paraffin, and sectioned at a thickness of 5 µm using a microtome. The prepared sections were stained using hematoxylin and eosin (H&E) for general histological evaluation. Each stained section was examined under an Olympus BX51 light microscope and documented using an Olympus C-5050 digital camera (Olympus Corporation, Tokyo, Japan) to capture representative images of pulmonary architecture.

#### 2.3.3. Histopathological Scoring Method

Lung injury was evaluated using a semi-quantitative scoring system modified from previously published methods. This system encompasses five key histopathological parameters: alveolar congestion (AC), hemorrhage (H), leukocyte aggregation within air spaces and vessel walls (AL), perivascular and interstitial edema (PE), and alveolar wall thickening and/or hyaline membrane formation (TA). Each parameter was assessed independently by a blinded pathologist and scored on a scale from 0 to 4, where 0 indicates no pathological involvement, 1 denotes minimal damage (≤25% of the field), 2 represents mild injury (26–50%), 3 reflects moderate damage (51–75%), and 4 corresponds to severe injury affecting more than 75% of the field. The individual scores were then summed to yield a total lung injury score, providing a comprehensive index of pulmonary damage severity [31].

### 2.4. Computed Tomography (CT) Imaging and Region of Interest (ROI) Analysis

Pulmonary computed tomography (CT) imaging was performed under deep anesthesia using a 16-slice multi-detector CT scanner (SOMATOM go.Now, Siemens Healthineers, Erlangen, Germany). The animals were placed in the supine position and carefully secured to the scanning table using soft restraints to minimize motion artifacts during image acquisition.

CT scans were obtained without contrast media. The scanning parameters included a tube voltage of 120 kV, variable mAs (automatic exposure control), 1 mm slice thickness, and a scan range from the C3 vertebra to the diaphragm, covering the entire lung field from apex to base. Acquired images were reconstructed at 1 mm slice thickness with no overlap, using a 512 × 512 matrix and a sharp reconstruction algorithm (Br64 kernel) to enhance spatial resolution of the lung parenchyma.

Quantitative assessment of pulmonary density was performed by measuring Hounsfield unit (HU) values. For each rat, six circular regions of interest (ROIs), each with a fixed area of 2.153 mm^2^, were manually placed on axial images at the level of the cardiac apex using lung window settings. Two ROIs were selected from each of the upper, middle, and lower zones of both lungs. Care was taken to avoid inclusion of major pulmonary vessels, bronchi, or osseous structures during ROI placement.

All CT images and HU measurements were independently evaluated by three radiologists who were blinded to the experimental group assignments and biochemical data. The average HU value from the six ROIs was calculated for each animal and used as the quantitative marker of pulmonary parenchymal density [25].

### 2.5. Arterial Blood Gas Analysis

Following thoracic CT imaging, 0.2 mL of arterial blood was collected from the right carotid artery of each rat using a 1 mL heparinized syringe. Arterial blood gas parameters (PaO_2_ and PaCO_2_) were measured immediately after collection using an automated blood gas analyzer (ABL800 FLEX, Radiometer Medical, Copenhagen, Denmark). All procedures were conducted under controlled room temperature and in compliance with standard preclinical sampling protocols.

### 2.6. Biochemical Analysis

At the end of the experimental period, blood samples were transferred into EDTA-containing tubes and centrifuged at 3000× rpm for 10 min at 4 °C to separate the plasma. The resulting supernatant (plasma) was carefully transferred to clean Eppendorf tubes and stored at −80 °C until biochemical analyses were performed.

#### 2.6.1. Measurement of Lipid Peroxidation

Plasma lipid peroxidation was assessed by measuring malondialdehyde (MDA) levels, an established indicator of oxidative stress. The assay was based on the reaction of thiobarbituric-acid-reactive substances (TBARS) with MDA, forming a chromogenic complex under acidic and high-temperature conditions. Plasma samples were mixed with trichloroacetic acid (TCA) to precipitate proteins, followed by the addition of TBARS reagent containing thiobarbituric acid (TBA). The mixture was vortexed and incubated at 100 °C for 60 min.

After incubation, samples were cooled on ice and centrifuged at 3000 rpm for 20 min. The absorbance of the supernatant was measured at 535 nm using a spectrophotometer. MDA levels (nM) were determined from a standard curve prepared with 1,1,3,3-tetramethoxypropane. This well-established method enabled sensitive detection of lipid peroxidation and oxidative plasma damage, as previously described by Placer et al. [32] and Ohkawa et al. [33].

#### 2.6.2. Measurement of TNF-α Levels

Plasma TNF-α levels were measured using a rat-specific ELISA kit (Abcam, Cat. No. ab236712, Cambridge, UK), validated for plasma and serum. The assay had a sensitivity of 1.04 pg/mL and a dynamic range of 18.75–1200 pg/mL. Absorbance was recorded at 450 nm. All samples were run in duplicate. According to the manufacturer, the intra-assay CV was 3.9%, and mean recovery in plasma was 82% (range: 80–83%), confirming assay precision and validity.

#### 2.6.3. Measurement of C-Reactive Protein (CRP) Levels

Plasma CRP levels were measured using a rat-specific sandwich ELISA kit (Abcam, Cat. No. ab108827, UK), validated for serum and plasma. The assay had a sensitivity of 0.2 ng/mL and a dynamic range of 0.391–25 ng/mL. Absorbance was measured at 450 nm. All samples were analyzed in duplicate. According to the manufacturer, intra- and inter-assay CVs were 4.3% and 9.8%, respectively, and the mean recovery in plasma was ~95%. Final concentrations were corrected for dilution.

#### 2.6.4. Measurement of Lactic Acid Concentrations

Plasma L-lactate concentrations were determined using a rat-specific competitive ELISA kit (MyBioSource, Cat. No. MBS755975, San Diego, CA, USA), validated for serum, plasma, and tissue homogenates. The assay had a detection range of 0.5–25 mmol/L and a sensitivity of 0.1 mmol/L. Absorbance was measured at 450 nm using a microplate reader. All samples were analyzed in duplicate, and intra-/inter-assay coefficients of variation were <10% and <12%, respectively. Final concentrations were calculated based on a four-parameter logistic standard curve.

#### 2.6.5. Measurement of Cyclic Guanosine Monophosphate (cGMP) Levels

Plasma cGMP levels were quantified using a competitive ELISA kit (Elabscience^®^, Cat. No. E-EL-0083, Houston, Texas, USA), validated for rat plasma samples. The assay had a sensitivity of 0.47 pmol/mL and a dynamic range of 0.78–50 pmol/mL. Absorbance was measured at 450 nm. All samples were analyzed in duplicate. According to the manufacturer, intra-assay CV was <10%, and recovery ranged from 80% to 120%, confirming the assay’s reliability and reproducibility.

#### 2.6.6. Measurement of Interleukin-6 (IL-6) Levels

Plasma IL-6 levels were measured using a rat-specific ELISA kit (R&D Systems, Cat. No. R6000B, Minneapolis, Minnesota, USA), validated for serum and plasma. The assay had a sensitivity of 36 pg/mL and a standard curve range of 62.5–4000 pg/mL. Samples exceeding the upper limit were diluted 1:10 with the kit-supplied buffer. Absorbance was measured at 450 nm, and all samples were run in duplicate. The intra-assay CV ranged from 4.5% to 8.8%, and the inter-assay CV from 7% to 10%, confirming analytical reliability.

#### 2.6.7. Measurement of Interleukin-1 Beta (IL-1β) Levels

Plasma IL-1β levels were measured using a rat-specific sandwich ELISA kit (MyBioSource, Cat. No. MBS825017, San Diego, CA, USA), validated for serum and plasma. The assay had a sensitivity of <15 pg/mL and a dynamic range of 62.5–4000 pg/mL. Absorbance was read at 450 nm using a calibrated microplate reader (Thermo Scientific Multiskan FC, Waltham, MA, USA). All samples were analyzed in duplicate. Intra- and inter-assay CVs were both below 10%, and final concentrations were calculated using a four-parameter logistic (4-PL) standard curve.

### 2.7. Statistical Analysis

All statistical analyses were performed using SPSS version 26.0 (IBM Corp., Armonk, NY, USA). The normality of data distribution was assessed using the Shapiro–Wilk and Kolmogorov–Smirnov tests, while homogeneity of variances was evaluated by Levene’s test.

Variables with normal distribution and homogeneous variances (e.g., MDA, TNF-α, CRP, cGMP, PaO_2_, PaCO_2_, CT-HUs) were analyzed using one-way analysis of variance (ANOVA). Tukey’s HSD test was applied for post hoc comparisons when variances were equal, whereas Tamhane’s T2 test was used when variances were unequal.

In contrast, non-normally distributed variables or those with unequal variances (e.g., IL-1β, alveolar congestion, hemorrhage, leukocyte aggregation, edema, wall thickening) were evaluated using the Kruskal–Wallis test, followed by Mann–Whitney U tests for pairwise comparisons. Bonferroni-adjusted significance thresholds were applied to correct for multiple comparisons where appropriate.

Data are expressed as mean ± standard error of the mean (SEM) for parametric variables and as median [interquartile range (IQR)] for non-parametric variables. A *p*-value of less than 0.05 was considered statistically significant.

## 3. Results

### 3.1. Statistical Power Analysis

To validate the adequacy of the sample size, post hoc power analyses were per-formed using the G*Power software (version 3.1.9.7). Across all measured end-points—including biochemical (e.g., MDA, IL-6, TNF-α, CRP), histological (e.g., alveolar congestion, hemorrhage, edema), radiological (CT Hounsfield units), and arterial blood gas (PaO_2_, PaCO_2_) variables—very large to extremely large effect sizes were observed (Cohen’s f = 1.03–4.17), with corresponding statistical power ranging from 95.4% to 99.9%. These findings confirm that the group sizes (*n* = 9–10) were sufficient to detect statistically and biologically meaningful differences.

### 3.2. Evaluation of Histological Lung Injury

#### 3.2.1. Alveolar Congestion (AC)

Alveolar congestion scores varied significantly among groups, as indicated by the Kruskal–Wallis test (χ^2^ = 32.179, *p* < 0.001). Median [IQR] values were markedly elevated in the FIP and FIP + Saline groups compared to control (*p* < 0.001), indicating pronounced vascular compromise. No difference was observed between FIP and FIP + Saline (*p* = 0.169). In contrast, MB significantly lowered congestion severity compared to FIP + Saline (*p* < 0.001), indicating vascular protective effects.

**Figure 2:**
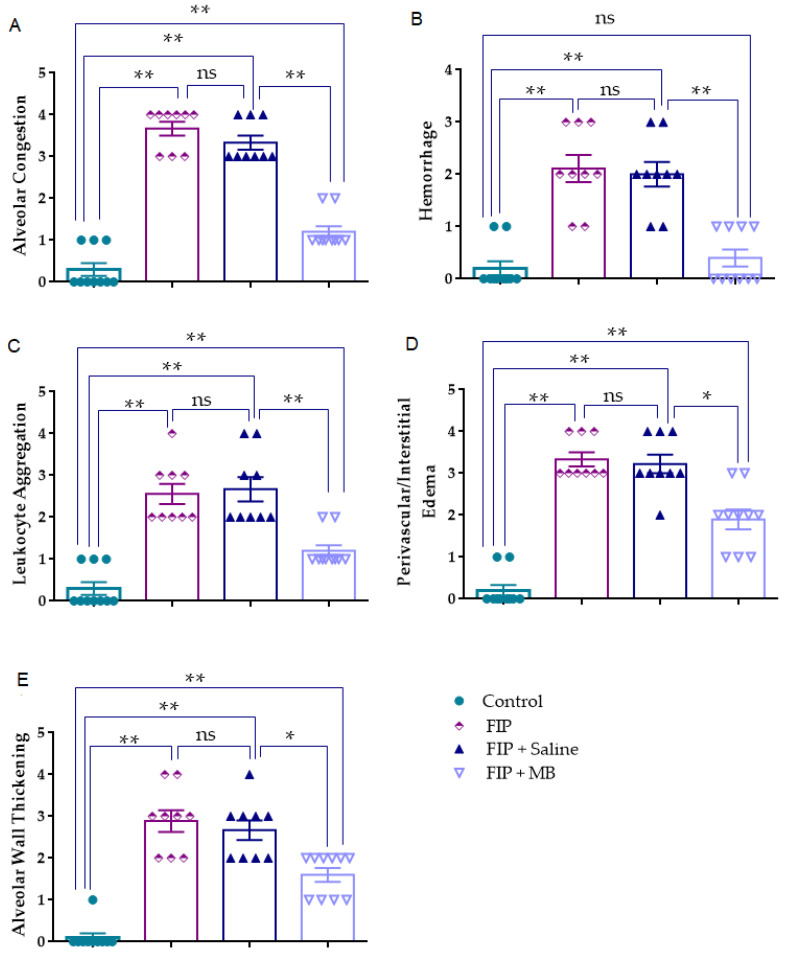
Histopathological scores of lung injury across experimental groups. (**A**) Alveolar congestion, (**B**) hemorrhage, (**C**) leukocyte aggregation, (**D**) perivascular/interstitial edema, and (**E**) alveolar wall thickening were evaluated semi-quantitatively. Data are presented as individual values with mean ± SEM. Statistical comparisons were performed using the Kruskal–Wallis test followed by Mann–Whitney U tests for pairwise analysis. * *p* < 0.01, ** *p* < 0.001, ns: not significant.

Moreover, alveolar congestion scores in the FIP + MB group remained significantly higher than those in the control group (*p* < 0.001), indicating only partial histopathological improvement (Figure 2, Table 1).

#### 3.2.2. Hemorrhage (H)

The severity of pulmonary hemorrhage differed significantly across experimental groups (Kruskal–Wallis χ^2^ = 26.590, *p* < 0.001). Median [IQR] scores were markedly elevated in the FIP and FIP + Saline groups compared to the control group (*p* < 0.001), reflecting the extent of vascular injury induced by FIP. However, no significant difference was observed between the FIP and FIP + Saline groups (*p* = 0.737), indicating that saline administration did not ameliorate hemorrhagic damage. MB treatment significantly reduced hemorrhage severity compared to the FIP + Saline group (*p* < 0.001). However, no statistically significant difference was observed between the FIP + MB and control groups (*p* = 0.3553), suggesting near-complete normalization of hemorrhage severity (Figure 2, Table 1).

#### 3.2.3. Leukocyte Aggregation (AL)

Leukocyte aggregation scores varied significantly among the experimental groups, as demonstrated by the Kruskal–Wallis test (χ^2^ = 29.600, *p* < 0.001). Median [IQR] values were markedly elevated in the FIP and FIP + Saline groups relative to the control group (*p* < 0.001 for both), indicating pronounced inflammatory cell infiltration following FIP induction. No significant difference was observed between the FIP and FIP + Saline groups (*p* = 0.844). In contrast, MB treatment significantly attenuated leukocyte accumulation compared to the FIP + Saline group (*p* < 0.001), reflecting its anti-inflammatory potential. Furthermore, leukocyte aggregation scores in the FIP + MB group remained significantly higher than those in the control group (*p* < 0.001), indicating only partial suppression of cellular infiltration (Figure 2, Table 1).

#### 3.2.4. Perivascular/Interstitial Edema (PE)

The severity of perivascular and interstitial edema differed significantly across the experimental groups, as determined by the Kruskal–Wallis test (χ^2^ = 29.776, *p* < 0.001). Median [IQR] scores were markedly elevated in both the FIP and FIP + Saline groups compared to the control group (*p* < 0.001), indicating pronounced microvascular leakage and fluid extravasation induced by the FIP model. No significant difference was observed between the FIP and FIP + Saline groups (*p* = 0.758). In contrast, MB treatment significantly reduced edema scores compared to the FIP + Saline group (*p* = 0.002), highlighting its role in mitigating endothelial dysfunction. Furthermore, edema scores in the FIP + MB group were significantly higher than in the control group (*p* < 0.001), indicating only partial reversal of vascular leakage (Figure 2, Table 1).

#### 3.2.5. Alveolar Wall Thickening (TA)

Alveolar wall thickening scores showed statistically significant differences among experimental groups, as indicated by the Kruskal–Wallis test (χ^2^ = 29.153, *p* < 0.001). Median [IQR] values were significantly elevated in the FIP and FIP + Saline groups compared to control (*p* < 0.001 for both), reflecting extensive interstitial inflammation and alveolar remodeling. No difference was observed between the FIP and FIP + Saline groups (*p* = 0.534). In contrast, MB treatment significantly reduced thickening scores compared to the FIP + Saline group (*p* = 0.006), indicating preservation of alveolar structure. Additionally, thickening scores in the FIP + MB group remained significantly higher than those in the control group (*p* < 0.001), reflecting only partial structural restoration (Figure 2, Table 1).

#### 3.2.6. Histopathological Evaluation of Pulmonary Tissue

**Figure 3:**
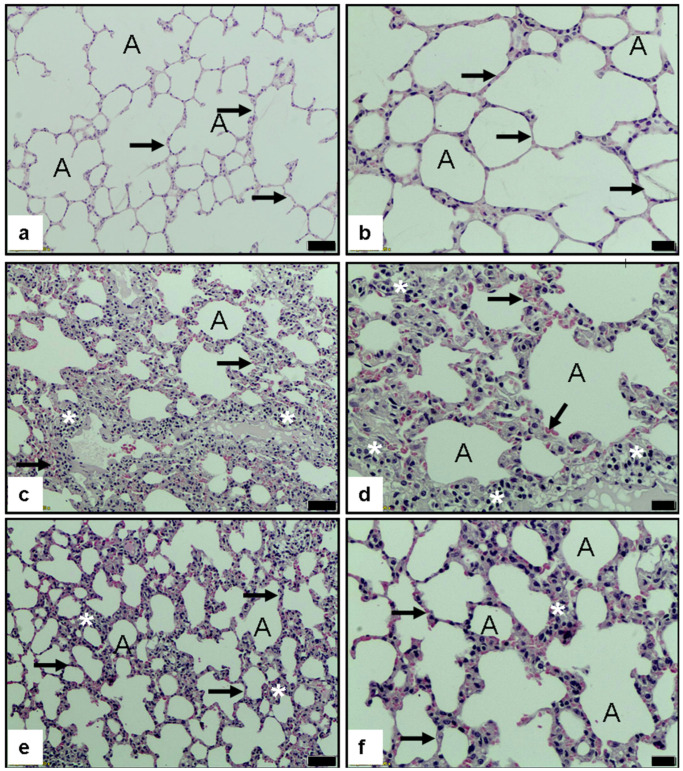
Lung histolopathology X 10 and X 20 magnification H&E staining. (**a**,**b**): normal control group lung alveola (A), (**c**,**d**): FIP and 10 mL/kg 0.9% NaCl saline (placebo) groups showed severe histopathologic alteration related to increased alveolar inflammation (*) and septal thickness (arrow), (**e**,**f**): FIP and 20 mg/kg methylene blue groups showed decreased inflammation and septal thickening (arrow) (scale bars are shown as 50 μm for 10 and 20 μm for 20 magnification).

Representative hematoxylin and eosin (H&E)-stained lung sections are shown in Figure 3. In the control group (a,b), the pulmonary architecture appeared intact, characterized by normal alveolar morphology (A), thin alveolar septa (black arrows), and well-preserved airspaces, with no evidence of inflammatory cell infiltration or parenchymal disruption.

In sharp contrast, the FIP + Saline group (c,d) exhibited severe histopathological damage consistent with acute lung injury. These changes included marked thickening of the alveolar walls (black arrows), dense leukocytic infiltration (white asterisks), and interstitial edema, indicating the presence of intense inflammatory and vascular injury induced by feces-induced peritonitis.

Remarkably, lung sections from the FIP + Methylene Blue (MB)-treated group (e,f) demonstrated substantial histological improvement. The alveolar walls were thinner, the extent of inflammatory cell infiltration was significantly reduced, and the overall alveolar architecture was largely preserved. Scattered minimal edema (*), along with reduced inflammatory foci, supported a partial restoration of tissue homeostasis. These findings indicate that MB exerts a protective effect on the lung, likely via its anti-inflammatory, antioxidant, and vascular-stabilizing properties.

### 3.3. CT-Based Assessment of Pulmonary Density Using Hounsfield Unit Measurements

**Figure 4:**
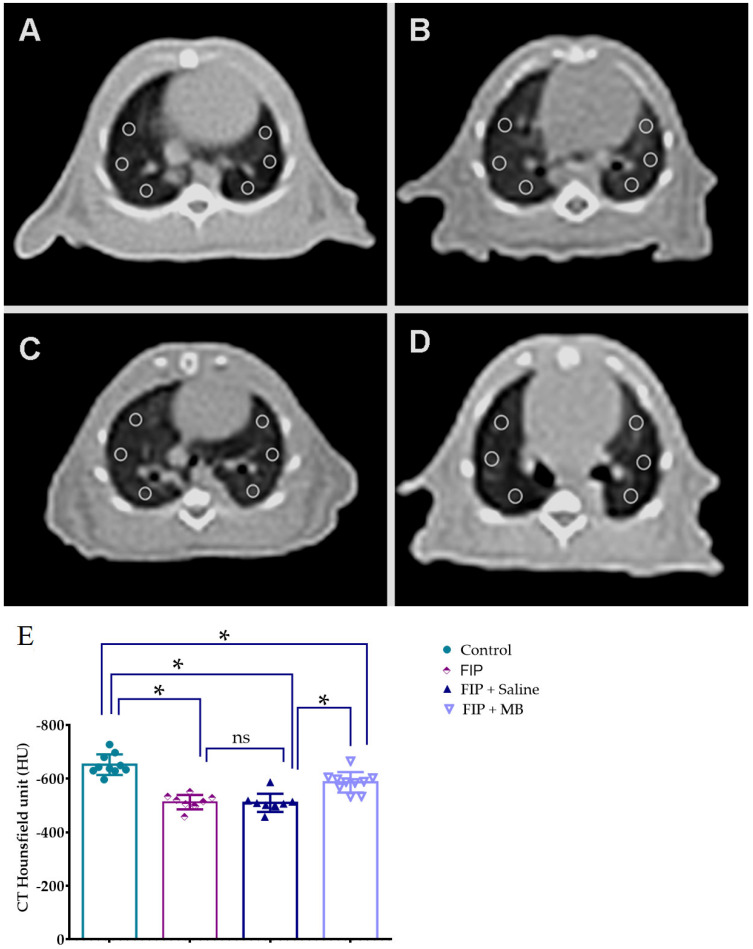
Axial lung CT images and quantitative analysis of parenchymal density. (**A**) Normal control group; (**B**) FIP gro up showing increased pulmonary density; (**C**) FIP + 0.9% NaCl (placebo) group showing similarly increased density; (**D**) FIP + MB group showing reduced density approaching normal. All images were taken at the cardiac level, with six identical ROIs placed at standardized locations. (**E**) Quantitative analysis of CT Hounsfield unit (HU) values. * *p* < 0.001. ns: not significant.

Computed tomography analysis revealed significant intergroup differences in lung density, as measured by Hounsfield units (HUs), with data satisfying the assumptions of normality and homogeneity (Kolmogorov–Smirnov *p* = 0.250; Levene’s test *p* = 0.758). One-way ANOVA demonstrated a significant overall group effect (F(3, 34) = 36.675, *p* < 0.001). HU values were significantly higher (less negative) in the FIP and FIP + Saline groups compared to control (*p* < 0.001 for both), indicating increased lung parenchymal density and reduced alveolar aeration due to inflammation and edema. No significant difference was observed between FIP and FIP + Saline groups (*p* = 0.998). In contrast, the FIP + MB group exhibited significantly lower HU values than both the FIP and FIP + Saline groups (*p* < 0.001 for both), indicating improved alveolar air content and attenuation of consolidation. Moreover, HU values in the FIP + MB group remained significantly higher than those in the control group (*p* < 0.001), suggesting partial radiological improvement (Figure 4, Table 2).

### 3.4. Arterial Blood Gas Analysis

#### 3.4.1. Arterial Oxygen Pressure (PaO_2_)

**Figure 5:**
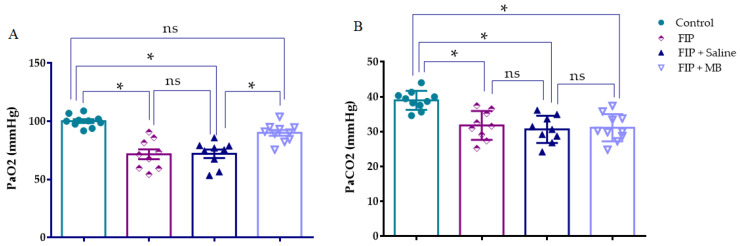
Arterial blood gas parameters in experimental groups. (**A**) PaO_2_ levels significantly decreased in the FIP and FIP + Saline groups co mpared to control, while MB treatment improved PaO_2_ compared to both groups (**B**) PaCO_2_ levels were significantly reduced in the FIP, FIP + Saline, and FIP + MB groups compared to control (* *p* < 0.001), with no significant differences observed in the FIP + Saline and FIP + MB groups. Data are presented as mean ± SEM. * *p* < 0.001; ns: not significant.

The distribution of PaO_2_ values satisfied both normality and homogeneity assumptions (Kolmogorov–Smirnov *p* = 0.779; Levene’s test *p* = 0.055). One-way ANOVA confirmed significant differences among the groups (F(3, 34) = 21.134, *p* < 0.001). PaO_2_ levels were significantly reduced in the FIP and FIP + Saline groups compared to control (*p* < 0.001 for both), indicating impaired alveolar oxygenation. No difference was observed between the FIP and FIP + Saline groups (*p* = 0.998), suggesting that isotonic saline failed to improve gas exchange. In contrast, MB treatment significantly increased PaO_2_ levels compared to both the FIP and FIP + Saline groups (*p* < 0.001 for both), indicating improved ventilation–perfusion matching. However, PaO_2_ levels in the FIP + MB group did not differ significantly from those in the control group (*p* = 0.0963), suggesting near-complete recovery of oxygenation capacity (Figure 5, Table 3).

#### 3.4.2. Arterial Carbon Dioxide Pressure (PaCO_2_)

PaCO_2_ values met normality and homogeneity assumptions (Kolmogorov–Smirnov *p* = 0.974; Levene’s test *p* = 0.530). One-way ANOVA revealed a significant group effect (F(3, 34) = 11.151, *p* < 0.001). PaCO_2_ levels were significantly reduced in the FIP and FIP + Saline groups compared to control (*p* < 0.001), suggesting increased respiratory drive or impaired CO_2_ diffusion. No significant difference was observed between the FIP and FIP + Saline groups (*p* = 0.915), indicating that saline had no effect on CO_2_ retention. Although PaCO_2_ levels appeared numerically improved in the FIP + MB group, no significant difference was detected compared to the FIP + Saline group (*p* = 0.993). However, PaCO_2_ levels in the FIP + MB group remained significantly lower than in the control group (*p* < 0.001), indicating persistent hyperventilation or compromised alveolar gas exchange (Figure 5, Table 3).

### 3.5. Assessment of Biochemical Parameters

#### 3.5.1. MDA (nM)

MDA levels followed a normal distribution (Kolmogorov–Smirnov *p* = 0.360), and Levene’s test confirmed variance homogeneity (*p* = 0.098). One-way ANOVA revealed a significant group effect (F(3, 34) = 58.010, *p* < 0.001).

**Figure 6:**
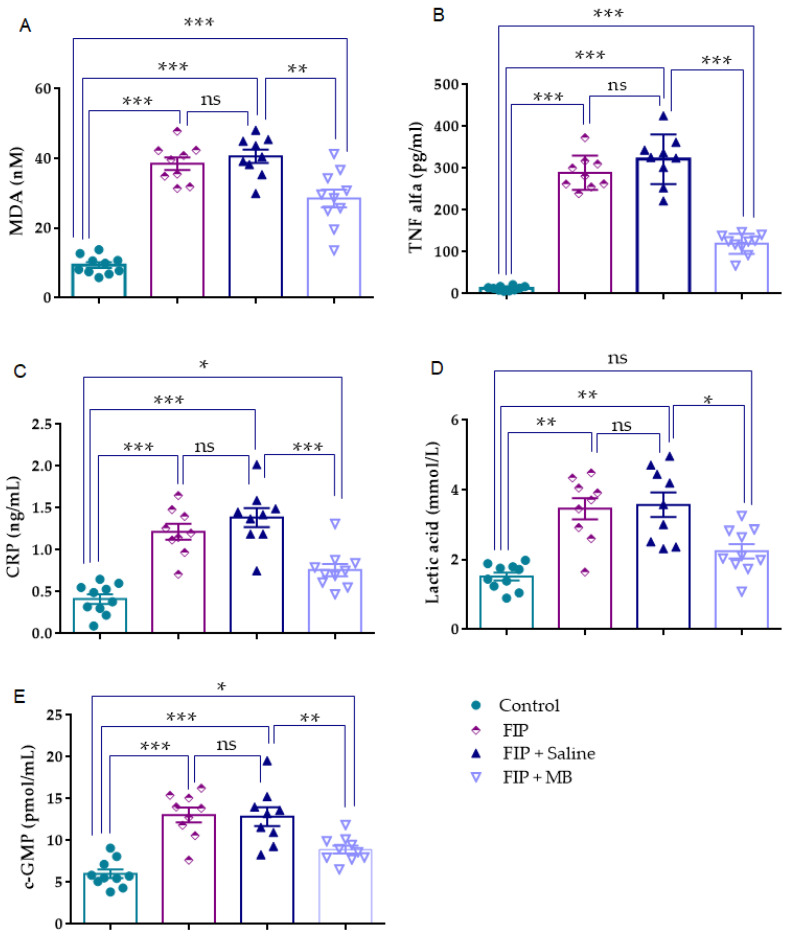
Biochemical alterations in FIP-induced lung injury and modulation by MB. (**A**–**E**) MDA, TNF-α, CRP, lactic acid, and cGMP levels were significantly increased in the FIP and FIP + Saline groups versus control, reflecting oxidative stress, inflammation, metabolic disturbance, and vascular activation. MB significantly reduced all markers. No significant differences were found between FIP and FIP + Saline groups. Data are mean ± SEM (*n* = 10). One-way ANOVA followed by Tukey’s post hoc test for MDA, CRP, and cGMP; Tamhane’s T2 for TNF-α and lactic acid. ns: not significant; * *p* < 0.05; ** *p* < 0.01; *** *p* < 0.001.

Both the FIP group and the FIP + Saline group showed significantly higher MDA levels compared to the control group (*p* < 0.001), indicating marked oxidative damage induced by FIP. The comparison between the FIP and FIP + Saline groups revealed no significant difference (*p* = 0.968). In contrast, MDA concentrations in the FIP + MB group were significantly reduced (*p* = 0.004 vs. FIP + Saline). Importantly, MDA levels in the FIP + MB group remained significantly higher than control (*p* < 0.001), indicating only partial restoration of oxidative balance (Figure 6, Table 4).

#### 3.5.2. TNF-α (pg/mL)

TNF-α levels conformed to the assumption of normality (Kolmogorov–Smirnov *p* = 0.394), although Levene’s test indicated heterogeneity of variances (*p* = 0.007). One-way ANOVA revealed a significant group effect (F(3, 34) = 145.207, *p* < 0.001), and Tamhane’s T2 post hoc test was applied.

Compared to the control group TNF-α levels were markedly elevated in both the FIP group and the FIP + Saline group (*p* < 0.001), indicating a strong systemic inflammatory response following FIP exposure. The comparison between the FIP and FIP + Saline groups showed no statistically significant difference (*p* = 0.267). In contrast, the FIP + MB group exhibited significantly lower TNF-α levels than both the FIP (*p* < 0.001) and FIP + Saline groups (*p* < 0.001). Additionally, TNF-α levels in the FIP + MB group remained significantly higher than those in the control group (*p* < 0.001), suggesting only partial attenuation of inflammation (Figure 6, Table 4).

#### 3.5.3. CRP (ng/mL)

CRP levels met the assumptions of normality and homogeneity of variances (Kolmogorov–Smirnov *p* = 0.634; Levene’s test *p* = 0.691). One-way ANOVA indicated a significant group effect (F(3, 34) = 26.924, *p* < 0.001). Both the FIP and the FIP + Saline groups (*p* < 0.001) exhibited significantly higher CRP concentrations than the control group, reflecting a robust acute-phase inflammatory response. No significant difference was observed between the FIP and FIP + Saline groups (*p* = 0.524). In contrast, CRP levels in the FIP + MB group were significantly lower than those in both the FIP and FIP + Saline groups (*p* < 0.001). Moreover, CRP levels in the FIP + MB group remained significantly elevated compared to the control group (*p* = 0.0274), indicating only partial resolution of the acute-phase response (Figure 6, Table 4).

#### 3.5.4. Lactic Acid (mmol/L)

Lactic acid levels were normally distributed (Kolmogorov–Smirnov *p* = 0.545), but Levene’s test indicated heterogeneity of variances (*p* = 0.013). One-way ANOVA showed a significant group effect (F(3, 34) = 15.408, *p* < 0.001), and Tamhane’s T2 post hoc test was applied. Both the FIP group (*p* = 0.001) and the FIP + Saline group (*p* = 0.002 vs. control) had significantly higher lactic acid levels than the control group, indicating increased anaerobic metabolism. No significant difference was observed between the FIP and FIP + Saline groups (*p* = 0.978). In contrast, the FIP + MB group showed significantly lower lactic acid levels compared to the FIP + Saline group (*p* = 0.036). However, no statistically significant difference was observed between the FIP + MB and control groups (*p* = 0.1803), indicating near normalization of lactic acid levels (Figure 6, Table 4).

#### 3.5.5. cGMP (pmol/mL)

Serum cGMP levels were normally distributed (Kolmogorov–Smirnov *p* = 0.913), and Levene’s test confirmed homogeneity of variances (*p* = 0.131). One-way ANOVA revealed a significant group effect (F(3, 34) = 19.156, *p* < 0.001). Compared to the control group, both the FIP and the FIP + Saline groups (*p* < 0.001) exhibited significantly elevated cGMP levels, suggesting nitric-oxide-driven activation of the guanylate cyclase pathway as part of FIP-induced endothelial dysfunction. No difference was observed between the FIP and FIP + Saline groups (*p* = 0.998). In contrast, MB administration significantly reduced cGMP concentrations compared to the FIP + Saline group (*p* = 0.005) (Figure 3, Table 1). Notably, cGMP levels in the FIP + MB group were also significantly lower than in the control group (*p* = 0.0490), suggesting a potential over-suppression of basal nitric oxide signaling (Figure 6, Table 4).

#### 3.5.6. IL-6 (pg/mL)

**Figure 7:**
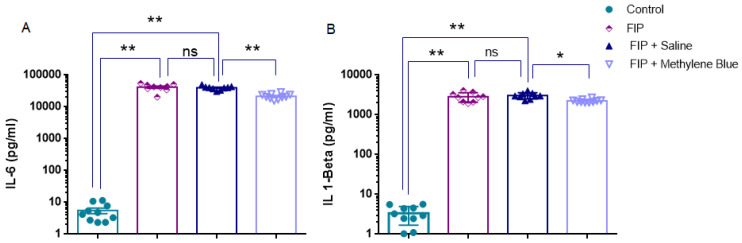
Effects of MB on IL-6 and IL-1β levels in FIP-induced inflammation. (**A**) Serum IL-6 and (**B**) IL-1β concentrations were significantly elevated in the FIP and FIP + Saline groups compared to control, indicating a pronounced inflammatory response. MB treatment led to a significant reduction in both cytokines, supporting its anti-inflammatory efficacy. No difference was observed between FIP and FIP + Saline groups. Data are presented as mean ± SEM. Y-axes are shown on a logarithmic scale. Statistical significance: ns, not significant; * *p* < 0.01; ** *p* < 0.001 (ANOVA with Tamhane’s T2 for IL-6; Mann–Whitney U for IL-1β). Data are presented as mean ± SEM. Y-axes are shown on a logarithmic scale. Note: logarithmic scale was applied to improve visualization of low values; however, it may underrepresent the absolute magnitude of change in high-value groups such as FIP + MB.

IL-6 levels satisfied the assumption of normality (Kolmogorov–Smirnov *p* = 0.171), although Levene’s test revealed heterogeneity of variances (*p* = 0.002). One-way ANOVA showed a highly significant group effect (F(3, 34) = 99.690, *p* < 0.001), and Tamhane’s T2 post hoc test was applied. IL-6 concentrations were markedly elevated in both the FIP and FIP + Saline groups (*p* < 0.001 vs. control), indicating a robust inflammatory response triggered by FIP-induced injury. No significant difference was observed between the FIP and FIP + Saline groups (*p* = 0.999). In contrast, MB administration significantly reduced IL-6 levels compared to FIP + Saline (*p* < 0.001). Additionally, IL-6 levels in the FIP + MB group remained significantly higher than in the control group (*p* < 0.001), indicating only partial resolution of inflammation (Figure 7, Table 5).

#### 3.5.7. IL-1β (pg/mL)

IL-1β levels followed a normal distribution (Kolmogorov–Smirnov *p* = 0.048); therefore, non-parametric analyses using the Mann–Whitney U test were applied. Compared to the control group, IL-1β concentrations were significantly elevated in both the FIP and FIP + Saline groups (*p* < 0.001), indicating a strong proinflammatory cytokine response. There was no significant difference between the FIP and FIP + Saline groups (*p* = 0.354), suggesting that saline treatment did not mitigate inflammation. In contrast, MB significantly reduced IL-1β levels compared to FIP + Saline (*p* = 0.001). Nevertheless, IL-1β concentrations in the FIP + MB group remained significantly higher than those in the control group (*p* < 0.001), indicating only partial cytokine suppression (Figure 7, Table 5).

## 4. Discussion

Sepsis is a life-threatening condition characterized by a dysregulated host response to infection, often culminating in ALI and multi-organ failure [3,34]. In the present FIP model of septic lung injury, MB demonstrated notable protective effects, attributed to its ability to suppress pro-inflammatory signaling and attenuate oxidative stress. These actions collectively contributed to preserved pulmonary structure and function, offering mechanistic insight into MB’s pleiotropic therapeutic potential.

As demonstrated in numerous studies, the FIP model triggers systemic inflammation secondary to intra-abdominal infection, frequently resulting in diffuse alveolar damage, interstitial edema, intra-alveolar hemorrhage, and neutrophilic infiltration in the lungs [22,25,35]. In our study, MB administration in FIP-induced rats significantly attenuated these pathological alterations, as reflected by lower composite lung injury scores indicating reduced edema, hemorrhage, inflammatory cell infiltration, alveolar congestion, and alveolar wall thickening. Notably, these histological improvements were accompanied by radiological and functional recovery. MB-treated rats exhibited lower lung parenchymal density on computed tomography (CT), indicating reduced consolidation and better aeration. Arterial blood gas analysis further confirmed enhanced oxygenation, with elevated PaO_2_ levels in the MB group compared to the untreated FIP group. These parallel improvements across microscopic, radiologic, and physiological parameters suggest that MB effectively alleviates alveolar inflammation and fluid accumulation. This aligns with previous studies where therapeutic interventions such as betaine in the study by Sorgun et al. [27], vitamins C and E [26], and oxytocin [25] yielded similar radiologic–histologic correlations in FIP-induced sepsis. In transfusion-related ALI, Song et al. [36] also demonstrated that treatment restored aerated lung volume and reduced diffuse damage, reinforcing the consistency of this relationship. Collectively, these findings support the validity of CT-derived lung density as a reliable non-invasive surrogate for histopathological severity in sepsis-related lung injury. The dual improvements observed in our study highlight the therapeutic potential of MB in mitigating pulmonary damage in FIP-induced sepsis.

Excessive NO production during sepsis—primarily via iNOS activation—stimulates sGC, leading to increased cGMP levels and vasoplegic shock [6,9,37,38]. This cascade results in vascular tone loss, impaired perfusion, and lactic acidosis [39,40,41]. In our study, FIP-induced rats showed elevated plasma cGMP and lactate levels alongside reduced PaO_2_, indicating compromised oxygen delivery. Notably, PaCO_2_ remained unchanged, suggesting selective impairment in oxygen exchange rather than ventilation. Arterial blood gases and lactate are well-established markers of tissue oxygenation and metabolic status. Accordingly, elevated lactate and decreased PaO_2_/FiO_2_ (arterial oxygen partial pressure to fraction of inspired oxygen ratio) have been associated with early mortality post-CLP [42], and similar trends have been reported in FIP models [25]. In our model, MB administration lowered lactate levels and improved PaO_2_, implying restoration of tissue perfusion. These findings are consistent with previous studies: Pan et al. [9] reported that methylene blue administration significantly reduced cGMP levels in a CLP-induced sepsis model, while Galili et al. [43] demonstrated that methylene blue preserved arterial oxygenation (PaO_2_) and improved hemodynamic parameters in rats subjected to CLP. Moreover, methylene blue has been shown to inhibit cGMP accumulation and enhance vasopressor responsiveness in various experimental models [44,45]. Taken together, these results suggest that MB may exert beneficial effects in septic states, potentially contributing to improved hemodynamic stability and pulmonary oxygenation, as supported by the observed reduction in cGMP levels.

Sepsis is characterized by a systemic cytokine storm that disrupts endothelial junctions and induces glycocalyx degradation, increasing vascular permeability and leading to pulmonary edema, as supported by multiple studies [41,46,47]. In parallel, oxidative stress—driven by the accumulation of reactive oxygen and nitrogen species such as peroxynitrite—contributes to the exacerbation of tissue damage [10,48,49]. Polymicrobial sepsis models corroborate these two key pathomechanisms. In both CLP and FIP, there is a marked elevation in pro-inflammatory cytokines such as TNF-α, IL-6, and IL-1β [9,25,50], along with increased MDA and MPO levels and reduced antioxidant defenses including SOD and GSH [16,51,52]. In line with these findings, our FIP model also exhibited significant elevations in IL-6, IL-1β, TNF-α, CRP, and MDA levels.

MB treatment significantly attenuated both pro-inflammatory cytokines and lipid peroxidation. These anti-inflammatory and antioxidant effects have been similarly reported in various pathological models, including colitis [53], ischemia–reperfusion injury [20], and diabetes [54], where MB administration reduced TNF-α, IL-1β, IL-6, and CRP levels, suppressed MPO and MDA, and enhanced SOD activity.

The pleiotropic anti-inflammatory effects of MB have been attributed to its inhibition of key signaling cascades, including NF-κB and the NLRP3 inflammasome, thereby preventing IL-1β maturation [55,56]. MB also suppresses IL-6/STAT3 (interleukin-6/Signal Transducer and Activator of Transcription 3) signaling and inhibits iNOS expression via modulation of the NO–sGC–cGMP pathway [57,58]. In our study, these mechanisms appear to be supported by the observed reductions in IL-6, TNF-α, CRP, and MDA, alongside a significant decrease in cGMP levels. Furthermore, histopathological findings were consistent with the suppression of inflammation, reinforcing the potential link between these biochemical and tissue-level effects. Taken together, our results suggest that MB may exert beneficial effects in sepsis through its anti-inflammatory and antioxidant properties, as evidenced by reductions in pro-inflammatory cytokines, oxidative stress markers, and cGMP levels.

However, the anti-inflammatory efficacy of MB has not been consistently observed across all studies, particularly in clinical settings. Notably, Memiş et al. [59] and Kirov et al. [60] reported no significant reductions in TNF-α or IL-6 levels following MB treatment. Similarly, Park et al. [61] found improved mean arterial pressure (MAP) without accompanying cytokine modulation. In a CLP model, Pan et al. [9] reported reduced cGMP levels after MB administration but no significant changes in TNF-α or IL-6. These discrepancies may reflect differences in sepsis severity or experimental design. Comparative studies indicate that the FIP model triggers a more intense inflammatory response than CLP, with markedly elevated IL-6 levels and more severe lung injury [21,22]. Another study further demonstrated that MB improved survival only in severe CLP cases, suggesting that its efficacy may depend on the intensity of systemic inflammation. These findings underscore the importance of disease severity and model selection in evaluating the therapeutic potential of methylene blue.

Taken together, the findings of this study indicate that methylene blue exerts marked anti-inflammatory, antioxidant, and vasoregulatory effects in feces-induced sepsis. These multifaceted properties translated into significant improvements in lung pathology, radiological parenchymal density, and arterial oxygenation. The application of the FIP model, combined with integrated biochemical, histological, and imaging-based assessments, underscores the methodological strength and translational relevance of this work. Given these results, methylene blue warrants further investigation as a potential therapeutic agent in sepsis-associated pulmonary injury, with additional studies needed to confirm its clinical relevance and determine optimal dosing strategies.

## 5. Conclusions

This preclinical study demonstrated that MB exerts protective effects in a FIP model of septic lung injury by attenuating inflammation, oxidative stress, and histopathological damage. The observed reductions in cGMP, pro-inflammatory cytokines, and lipid peroxidation markers suggest that MB may modulate multiple injury pathways involved in sepsis pathophysiology. Improvements in gas exchange and radiologic lung findings further indicate its potential to preserve pulmonary function. However, these findings are preliminary and limited to an acute, single-dose, preclinical model. Further studies are necessary to define its pharmacodynamic profile, evaluate safety, and investigate its effectiveness in combination with standard sepsis therapies.

### Limitations

This study has several limitations. First, although MB was administered one hour after FIP induction, no dose–response or time–response analyses were conducted. As such, the optimal therapeutic timing and dosage remain undefined. Moreover, potential adverse effects were not evaluated; although no clinical signs of toxicity were observed, comprehensive safety assessments are warranted to confirm tolerability under septic conditions [5].

Second, vascular permeability and endothelial integrity were not assessed via tight junction staining, which limits mechanistic interpretation. The study also focused solely on short-term inflammatory and histological outcomes, without evaluating long-term endpoints such as survival or fibrosis.

Third, the exclusive use of male rats limits generalizability. Studies have demonstrated sex-related differences in sepsis outcomes, with evidence indicating that hormonal and physiological fluctuations associated with the estrous cycle in females may introduce variability into experimental data [62,63]. Kennedy et al. [64] reported significantly higher WBC and monocyte counts at 12 h in metestrus versus estrus, with a trend toward increased mortality. Similarly, Zellweger et al. [65] observed enhanced immune tolerance during proestrus. While Sharma et al. [66,67] used both sexes in the FIP model, their endpoints were assessed at 72 h. In contrast, our 24 h design is more sensitive to early hormonal variation. Therefore, male rats were selected to minimize variability. Future studies should incorporate female cohorts to assess sex-specific responses.

Fourth, the absence of a positive control group is a limitation. However, this was ethically justified under the 3R principles, as this study represents the first evaluation of MB at 20 mg/kg (i.p.) in the FIP model. Including a standard therapy without prior efficacy data would have resulted in unnecessary animal use. Based on encouraging results, future studies will include positive and sham/placebo control groups to contextualize MB’s efficacy.

Lastly, although ketamine/xylazine anesthesia may affect inflammatory and NO–cGMP pathways at higher doses or later time points, we used a low-dose regimen (40/4 mg/kg) with rapid blood sampling (~10 min) to minimize such effects. Previous studies indicate that cytokine alterations occur several hours post-anesthesia [68], NO increases begin around 15 min [69] and cGMP elevations at 60 min [70]. Additionally, key metabolic markers (MDA, GSH, lactate) remain stable within this early window. Nonetheless, further evaluation of dose and timing effects is recommended.

## Figures and Tables

**Table 1:** Histopathological scores of lung injury parameters across experimental groups.

	Control	FIP	FIP + Saline	FIP + MB
AC	0.0 [0.0–1.0]	4.0 [3.0–4.0] *	3.0 [3.0–4.0] *	1.0 [1.0–1.25] *^,##^
H	0.0 [0.0–0.25]	2.0 [1.5–3.0] *	2.0 [1.5–2.5] *	0.0 [0.0–1.0] ^##^
AL	0.0 [0.0–1.0]	2.0 [2.0–3.0] *	2.0 [2.0–3.5] *	1.0 [1.0–1.25] *^,##^
PE	0.0 [0.0–0.25]	3.0 [3.0–4.0] *	3.0 [3.0–4.0] *	2.0 [1.0–2.25] *^,#^
TA	0.0 [0.0–0.0]	3.0 [2.0–3.5] *	3.0 [2.0–3.0] *	2.0 [1.0–2.0] *^,#^

Values are presented as median [IQR]. Kruskal–Wallis test with Mann–Whitney U post hoc comparisons. * *p* < 0.001 vs. control; ^#^ *p* < 0.01, ^##^ *p* < 0.001 vs. FIP + Saline group.

**Table 2:** Computed tomography (CT) Hounsfield unit (HU) values in experimental groups.

	Control	FIP	FIP + Saline	FIP + MB
CT Hounsfield unit (HU)	−652 ± 12.13	−512 ± 8.9 *	−509 ± 11.27 *	−586 ± 12.06 *^,#^

Values are mean ± SEM. ANOVA with Bonferroni’s post hoc test. * *p* < 0.001 vs. control; ^#^ *p* < 0.001 vs. FIP + Saline.

**Table 3:** Arterial oxygen (PaO_2_) and carbon dioxide (PaCO_2_) pressures in experimental groups.

	Control	FIP	FIP + Saline	FIP + MB
PaO_2_ (mmHg)	100.2 ± 1.645	71.64 ± 4.219 *	72.09 ± 3.614 *	90.04 ± 2.473 ^#^
PaCO_2_ (mmHg)	38.98 ± 0.8624	31.78 ± 1.379 *	30.65 ± 1.293 *	31.12 ± 1.237 *

Values are mean ± SEM. ANOVA with Bonferroni’s post hoc test. * *p* < 0.001 vs. control; ^#^ *p* < 0.001 vs. FIP + Saline.

**Table 4:** Effects of MB on oxidative stress, inflammatory cytokines, metabolic indicators, and vascular signaling parameters in FIP-induced lung injury.

	Control	FIP	FIP + Saline	FIP + MB
MDA (nM)	9.3 ± 0.8	38.52 ± 1.8 ***	40.62 ± 1.8 ***	28.53 ± 2.5 ***^,##^
TNF alfa (pg/mL)	12.56 ± 1.61	289.1 ± 13.69 ***	321.7 ± 19.80 ***	119.3 ± 7.646 ***^,###^
CRP (ng/mL)	0.4 ± 0.05	1.2 ± 0.09 ***	1.3 ± 0.11 ***	0.7 ± 0.07 *^,###^
Lactic acid (mmol/L)	1.5 ± 0.1	3.4 ± 0.3 **	3.5 ± 0.3 **	2.2 ± 0.2 ^#^
c-GMP (pmol/mL)	6.01 ± 0.5	13.06 ± 0.9 ***	12.87 ± 1.12 ***	8.9 ± 0.47 *^,##^

Results are presented as mean ± SEM. Statistical analyses were performed by one-way ANOVA. * *p* < 0.01, ** *p* < 0.01, *** *p* < 0.001 different from normal groups; ^#^ *p* < 0.05, ^##^ *p* < 0.01, ^###^ *p* < 0.001 different from FIP + Saline group.

**Table 5:** Effects of MB on inflammatory cytokines parameters in FIP-induced lung injury.

	Control	FIP	FIP + Saline	FIP + MB
IL-6 (pg/mL)	5.5 ± 1.07	41,523 ± 3406 *	39,864 ± 1836 *	21,612 ± 1294 *^,##^
IL 1-Beta (pg/mL)	3.34 ± 0.52	2520 ± 185.1 *	3052 ± 177.7 *	2244 ± 70.06 *^,#^

Results were presented as mean ± SEM. Statistical analyses were performed by one-way ANOVA. * *p* < 0.001 different from normal groups; ^#^ *p* < 0.01, ^##^ *p* < 0.001 different from FIP + Saline group.

## Data Availability

Data are available on request due to ethical/privacy reasons.
